# Curricular Redundancy in Medical Undergraduate Course: Critical Insights from Various Stakeholders in Medical Institutes in India

**DOI:** 10.1055/s-0044-1791843

**Published:** 2024-10-28

**Authors:** Pradip B. Barde, Naresh Parmar, Vinay Chitturi, Gaurav Sharma, Rajesh Kathrotia, Krupal Joshi, Manisha Naithani, Vivek Kumar Sharma

**Affiliations:** 1 Department of Physiology, All India Institute of Medical Sciences, Rajkot, Gujarat, India; 2 Department of Community and Family Medicine, All India Institute of Medical Sciences, Rajkot, Gujarat, India; 3 Department of Biochemistry, All India Institute of Medical Sciences, Rishikesh, Uttarakhand, India

**Keywords:** curricular redundancy, medical undergraduate, new content

## Abstract

**Background** Medical education is continuously evolving to keep pace with the dynamic field of medicine. This study addresses the issue of curricular redundancy in medical education, highlighting the necessity for periodic reviews to eliminate outdated or irrelevant topics.

**Method** A descriptive qualitative approach was employed, involving participants from various medical schools across India. An online questionnaire was used to gather data on redundant topics, suggested replacements, and improvements in the curriculum for both didactic and non-didactic subjects, along with inputs related to assessment and evaluation methods. Content analysis was used for thematic identification and qualitative interpretation.

**Results** Out of 71 respondents from a wide geographic distribution, 30% were female, and 70% were male, with an age range of 17 to 36 years. Participants expressed concerns about redundant theoretical (32%) and practical (51%) topics. Qualitative analysis highlighted the need for integrating different subjects and placing a stronger focus on practical clinical skills. Participants emphasized the importance of a curriculum that keeps pace with advancements in medicine, such as genomics and artificial intelligence, while also addressing mental health. Specifically, they suggested combining anatomy and surgery courses, incorporating more active learning techniques, and utilizing ongoing assessments to gauge progress.

**Conclusion** The study highlights the necessity of eliminating curricular redundancy in medical education. Recommendations include developing a flexible curriculum, emphasizing region-specific content, and implementing a formative assessment system. Additionally, the importance of faculty development and stakeholder involvement in curriculum design is emphasized.

**Key Message**

Key insights for updating the medical undergraduate curriculum include the following:

Developing a flexible curriculum.

Emphasizing region-specific content.

Implementing formative assessments.

## Introduction

There is a pressing need for continuous curriculum evolution in medical education as it is a dynamic field that must adapt to the ever-changing landscape of medicine. With new knowledge and practices emerging constantly, medical school curricula require regular updates to stay current. 1 Simultaneously, maintaining a streamlined curriculum is crucial. This involves identifying and removing redundant topics to ensure efficiency and clarity.

Curricular redundancy is a significant challenge for any educational program, as it wastes time and resources, making it difficult for students to focus on essential topics. Redundant topics can also add complexity, hindering navigation through the curriculum. 2

Several factors contribute to curricular redundancy. One is the persistence of outdated or irrelevant information, often included due to tradition rather than current relevance. 3 Another factor is the repetition of the same topic across different courses, albeit from varying perspectives.

Addressing redundancy requires a collaborative approach, involving regular curriculum reviews and feedback from both students and faculties. 4 Research on curricular redundancy in medical education has gained importance. Studies suggest that up to 20% of medical school curricula may be redundant, 5 and surveys show that both students and faculties agree on the need to eliminate such content. 6

The negative impact of curricular redundancy on student learning is well-documented. Studies indicate that students exposed to redundant content struggle with recall and application of information, 3 and this exposure can lead to burnout. 4 While student-led studies have explored redundancy in highly integrated curricula, 7 comprehensive feedback from various stakeholders remains limited in the literature.

In response to these concerns, a qualitative study was designed to gather feedback from diverse stakeholders on the issue of curricular redundancy. The aim is to identify and address redundancy by exploring alternative approaches and replacements for redundant curriculum content.

## Materials and Methods

We employed a descriptive qualitative approach to investigate the perspectives of medical faculties, residents, interns, and students regarding the medical undergraduate curriculum in India. The participants were included from various medical schools across India. We used a random block sampling method to select participants from colleges in different regions, encompassing both rural and urban areas, and included faculty members, final-year undergraduate, and postgraduate students.

Data collection was conducted using a pretested and prevalidated online questionnaire consisting of 11 items, including open-ended questions. The questionnaire was distributed electronically, with informed consent obtained prior to participation. Individual responses were collected without personal identifiers, compiled, and anonymized before analysis to ensure confidentiality.

The questionnaire was structured as follows: the first five questions gathered demographic and general information. The subsequent section asked participants to identify topics or competencies in the medical undergraduate curriculum they deemed redundant or unnecessary and to provide a rationale for their opinions. The next part of the questionnaire focused on suggestions for replacement, modification, or new topics to be included in the curriculum. The final section addressed necessary changes in the curriculum pertinent to the undergraduate exit exam or postgraduate entrance exam, including potential modifications in assessment methods.

The collected descriptive data were compiled into a transcript. We employed content analysis, a qualitative research method, to identify patterns and themes within the qualitative data. The content analysis involved several steps: data reduction, coding, theme identification, and qualitative interpretation. Additionally, we conducted a thematic analysis of the qualitative data to reveal patterns, themes, and insights that addressed the research question and met the research objectives.

## Results

**Fig. 1:**
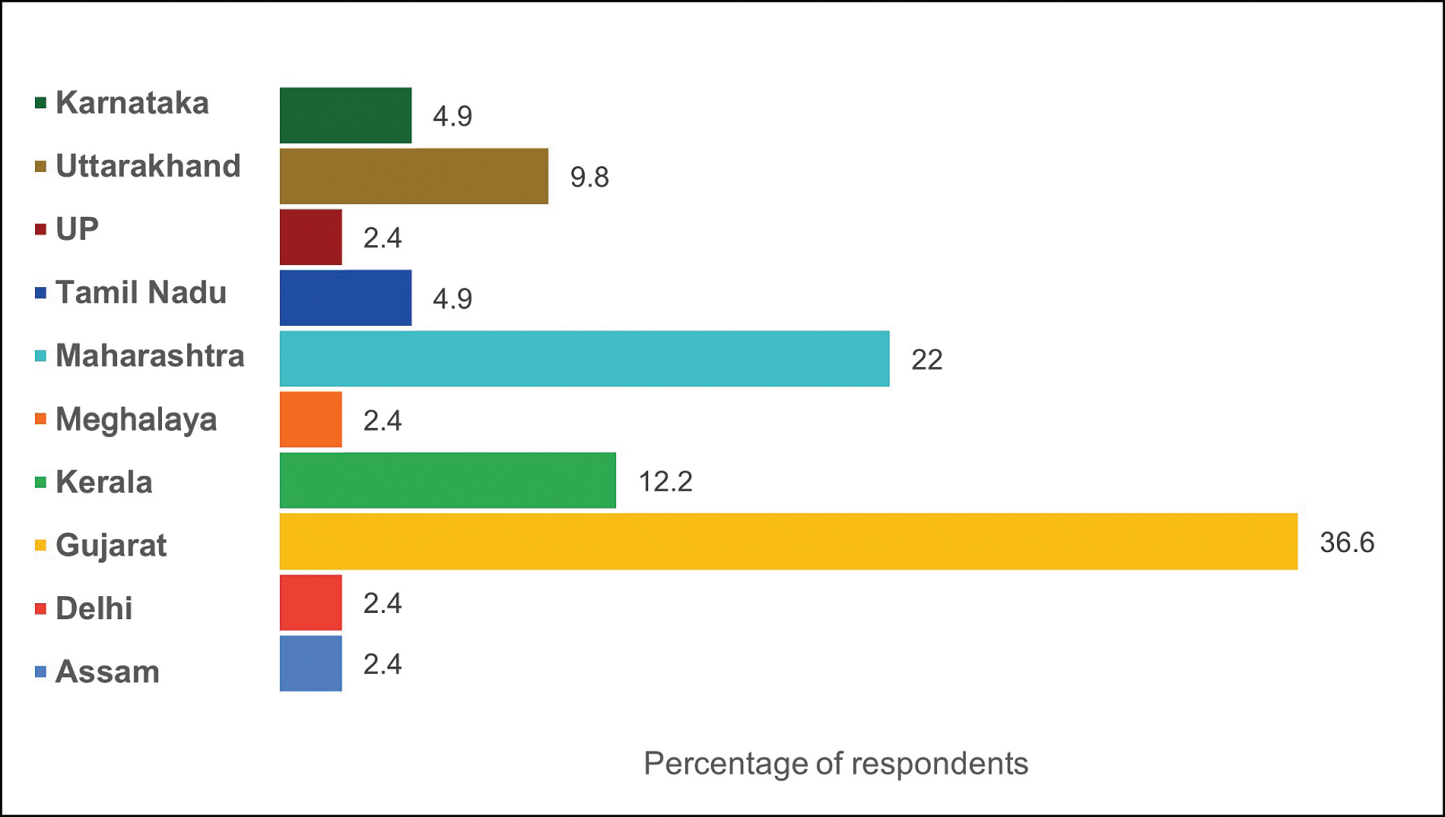
Geographic (state-wise) distribution of participants in India.

A total of 71 participants responded and completed the Google form. Among them, 21 (30%) were female and 50 (70%) were male, with a median age of 27 years (ranging from 17 to 40 years). The participants included faculties (63%), residents (10%), and students and interns (27%). The medical education training background of the faculty participants varied from essential to advanced medical education certification. The geographic distribution of the participants covered almost all regions of India ( Fig. 1 ).

**Fig. 2:**
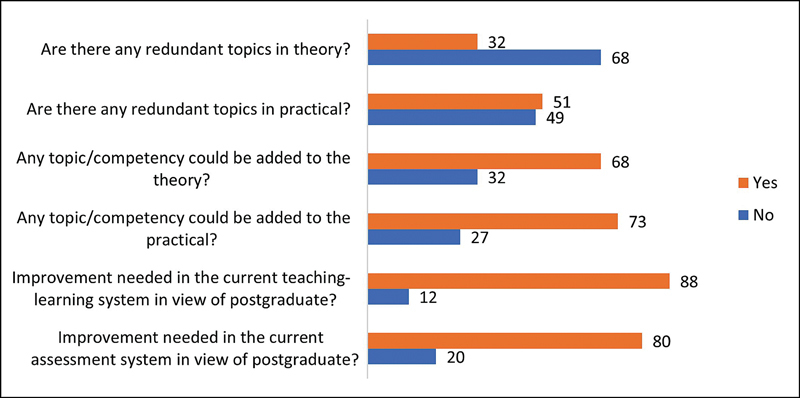
Participants' responses regarding redundancy and inclusion of new theory and practical curriculum topics.

The identification of redundant topics and the need for the inclusion of new topics in the theoretical and practical curriculum, as per responses received from the participants, are shown in Fig. 2 .

The qualitative data analysis with identified themes is compiled in Table 1 . Significant suggestions are briefly mentioned. The questions regarding the content analysis of input about preexisting theory curriculum topics (didactic teaching) highlighted the need for flexibility to incorporate evolving medical knowledge. Participants suggested integrating basic sciences with clinical subjects for better understanding, rather than eliminating obsolete topics entirely. Many proposed retaining an overview of specific outdated topics for historical perspective. Both students and faculties emphasized the need for better and more effective integration. Suggestions included moving topics like “function of hormones/endocrinology” and premedical (basic science) topics such as “general immunology” and “metabolism of carbohydrates, fats, and proteins” to other relevant subjects.

**Table 1:** Depicting key theme areas identified and primary suggestions given by participants

Serial number	Theme area identified	Major suggestions
1	Content analysis of input about preexisting theory curriculum topics (didactic teaching)	• Providing only an overview of specific obsolete topics for a historical perspective • Combining anatomy and surgery • Integrating subjects like physiology, pharmacology, pathology, and medicine more cohesively
2	Content analysis of input about Inclusion of new theory curriculum topics (didactic teaching)	• Public health and emerging infections: with a focus on learning about the pandemic of COVID-19 • Hospital management and administration • Incorporation of newer technology: bioinformatics, robotics, genetics, and artificial intelligence • Introduction to newer specialized fields of medicine: geriatrics, neuropsychiatry, fetomaternal medicine, different specialties in dentistry • Focus on mental health • Antibiotic policies • Finances • Addition of new topics such as “brain death,” “perimenopause,” “treatment of infertility,” “accreditation process,” and “diet of Indian patient” • Focus on practical implications of communication skills and the doctor–patient relationship • Yog or similar traditional holistic practices and approaches with scientifically proven and clearly indicated in the management given therapy area
3	The content analysis of feedback on the existing nondidactic teaching curriculum (laboratory-based practical/clinical skills)	• Reducing lecture classes and introduction of more practical/skill-based training • Eliminate outdated content: – Hematology techniques, such as Hb by Sahli's method and total RBC and WBC count by Neubauer's chamber – “Mosso's ergography” experiment – Urine-based testing using older outdated methods – Amphibian and animal experiments – Pharmacy practical • Stricker enforcement of clinical rounds • Exposure to real-world patient care settings • Integration of AI and automation
4	A content analysis of inputs about new curriculum topics for nondidactic teaching (laboratory practical/clinical postings) revealed several key themes	• Improved posting with emphasis on hands-on training and practical skills, preanalytical errors affecting sample collection and testing, laboratory report interpretation, computer-assisted case-based scenarios with focus on clinical trial, PK calculations, and case studies with medicolegal importance • Posting time to be utilized for direct patient interaction rather than solely focusing on theoretical concepts • Practical management of trauma, acute conditions, emergency management
5	The areas of improvement needed in the current teaching–learning system	• Utilizing flipped classroom • Emphasis on vertical integration • More practical skill training • More inclusion of hands-on experiences • Encouraging self-paced and self-directed learning • Introduce near-peer and peer-to-peer learning sessions
6	The areas of improvement needed in the current assessment system for undergraduates in the context of postgraduate and superspecialty entrance examinations	• Inclusion of MCQ-based assessment to mimic the pattern of postgraduate entrance examinations • Stricter assessments of clinical and surgical skills • Include various assessment methods: case scenario-based assessment, OSCE/OSPE, applied and clinical questions, clinical vignettes, image and video-based MCQs, Mini-CEX, and workplace-based assessments.

Abbreviations: AI, artificial intelligence; Hb, hemoglobin; MCQ, multiple choice question; Mini-CEX, mini-clinical evaluation exercise; OSCE, objective structured clinical examinations; OSPE, objective structured practical examinations; PK, pharmacokinetic; RBC, red blood cell; WBC, white blood cell.

Content analysis of input about the inclusion of new theory curriculum topics (didactic teaching) indicated a clear mandate to address current clinical scenarios. There were recommendations to adapt by adding topics that would benefit students, such as the impact of the COVID-19 pandemic. Many respondents suggested including topics related to emerging public health threats, such as disease epidemiology, biostatistics, and health economics.

The content analysis of feedback on the existing nondidactic teaching curriculum, including laboratory-based practical/clinical skills, revealed suggestions to remove outdated content in practicals while retaining clinically relevant exercises like the peripheral blood smear. The opinion emerged that redundant practical topics should be offered as demonstration exercises or made optional for students with specific interests. Respondents emphasized the importance of hands-on experience in solidifying theoretical knowledge and developing practical skills. They proposed introducing more skill laboratory sessions, providing students with dedicated time to practice and refine their clinical skills under the guidance of experienced instructors. The need to review the current curriculum was also highlighted to adequately prepare students for contemporary pharmacy practices. Emphasis on clinical experience underscores the critical role of practical training in preparing future medical professionals for the challenges and complexities of their careers.

A content analysis of inputs about new curriculum topics for nondidactic teaching (laboratory practical/clinical posting) revealed several key themes: Participants highlighted the need for competency-based training in laboratories or clinical postings. They suggested that these postings could be used to provide exposure to practical applications of theoretical concepts, such as body composition analysis and obesity assessment, training in nerve conduction studies, electromyography, brain evoked response auditory, advanced critical life support, ventilator use, ultrasound-guided procedures, portable echocardiography, and point-of-care ultrasound. Skill-enhancing short courses on doctor–patient relationships, communication, ethics, and medicolegal topics were recommended to improve healthcare and address the rising incidence of violence against doctors and healthcare team members.

Participants also suggested improvements in the current teaching–learning system, emphasizing active learning. They recommended more problem-based learning techniques, flipped classrooms, and small group discussions focused on real clinical cases. Further suggestions included increased practical skill training, hands-on experiences, and an emphasis on conceptual learning through laws, principles, charts, and graphs.

Regarding improvements needed in the current assessment system for undergraduates, particularly in the context of postgraduate and superspecialty entrance examinations, there was a clear emphasis on clinical skills and practice. Respondents suggested that medical students should be prepared to become better doctors, rather than just focusing on entrance examinations. Implementing stricter practical-based assessments to encourage the achievement of competencies was a recurring suggestion.

## Discussion

Redundancy in the medical curriculum can act as a double-edged sword, with both positive and negative impacts depending on the specific topics and the type of curriculum adopted. 7 Participants' views on existing and new theoretical curriculum topics in didactic teaching provide valuable insights from educators, medical professionals, and stakeholders in medical education. The theme of “Redundant Topics and Their Relevance” highlights concerns about redundancy in the curriculum. Some competencies were deemed unnecessary for undergraduate medical students, while others were considered outdated and irrelevant. Redundancy in teaching topics across multiple subjects is a common issue in medical education. Redundant topics can overload students, hindering their ability to effectively absorb and apply essential knowledge. Researchers have emphasized the need for curriculum revision to eliminate redundancy and streamline content to meet the evolving demands of medical practice. 8 The subtheme of transferring specific competencies to more specialized areas aligns with the concept of specialization and the recognition that medical education should prepare students for their chosen specialties. 9 10

The subtheme of “the forward-looking curriculum” proposes the inclusion of specific topics in future years and emphasizes the need for flexibility and adaptability in the curriculum, aligning with a competency-based and lifelong learning approach in medical education. 11 The evolving nature of medical knowledge necessitates an adaptable curriculum to ensure that graduates remain up-to-date with current practices. 12

Regarding the subtheme of “shifting/integrating topics between subjects,” participants suggested moving specific topics to more appropriate subjects and integrating molecular biology into biochemistry. This reflects ongoing discussions in medical education about the need for interdisciplinarity. 13 Medical education increasingly recognizes the importance of breaking down traditional subject silos to enhance knowledge integration. The subtheme of “pedagogical considerations” highlighted participants' preference for traditional teaching methods like “chalk and board” and emphasized practical and clinical components. This underscores the ongoing debate in medical education about the most effective teaching methods. Some educators advocate for a balance between traditional didactic teaching and active learning approaches. 14 The importance of practical and clinical components is also well-established. 15 Furthermore, the subtheme of “comprehensive integration of clinical subjects” suggests merging anatomy and surgery, and integrating subjects such as physiology, pharmacology, pathology, and medicine, reflecting the ongoing trend of interdisciplinary and integrated medical curricula. 16 Integration helps bridge the gap between basic science knowledge and clinical practice.

In terms of changes in teaching–learning methods, there is a suggestion to provide an overview of obsolete topics for historical context rather than excessive detail, aligning with principles of curricular efficiency. Researchers argue that a leaner curriculum focused on core competencies can enhance students' retention and application of knowledge. 17 Inputs for integrating emerging topics reflect the need to keep medical education aligned with rapid advancements in medical science and technology. 18 These emerging topics, including epigenetics, genomics, proteomics, bioinformatics, and artificial intelligence, are increasingly relevant in modern medicine. Emphasizing the doctor–patient relationship and communication highlights the importance of professionalism and interpersonal skills in medical practice. 19 Effective communication improves patient outcomes and satisfaction. 20 Recognizing the importance of mental health and well-being in the curriculum acknowledges the growing awareness of mental health challenges faced by medical students and practitioners. This aligns with recommendations for mental health support and stress management in medical education. 21 Including topics related to hospital management and administration reflects the recognition that healthcare professionals need a comprehensive understanding of healthcare systems and administrative processes to provide high-quality care. 22 23 This is essential in an era of increasingly complex healthcare delivery.

Regarding nondidactic topics, respondents expressed the need to eliminate or make specific redundant topics optional, such as manual estimation of hemoglobin, red blood cell, and white blood cell counts. Emphasis should be given to the introduction of modern techniques. 5 This suggests that some medical schools may still be teaching outdated manual methods despite the availability of more modern and efficient techniques. Respondents emphasized aligning the curriculum with clinical practice needs and removing nonessential components. 4 This indicates that some medical schools may teach irrelevant or nonessential topics, wasting students' time and preventing them from learning the necessary skills and knowledge for clinical practice. Respondents also stressed the need to streamline and simplify the curriculum, focusing on logical understanding over rote memorization. 3 This suggests that some medical schools may overload students with information and rote memorization, leading to burnout and decreased knowledge retention. Emphasizing precise and safe medication administration underscores the importance of this essential skill for healthcare practitioners. 24

In terms of newer topics in didactic and nondidactic learning, the focus on clinical and practical learning aligns with principles of problem-based learning (PBL) and competency-based medical education (CBME). These approaches prioritize real-world clinical experiences and hands-on learning to prepare students for the demands of medical practice. Several studies have demonstrated the effectiveness of PBL and CBME in enhancing clinical reasoning and practical skills among medical students. A review highlighted the positive impact of PBL on clinical skills and knowledge acquisition. 25

Recommendations for reducing lecture-based classes and incorporating active learning methods such as small group discussions and self-directed learning align with modern principles of active learning and learner-centered education. Research in medical education has shown that active learning strategies, such as problem-based learning, team-based learning, and case-based learning, improve learning outcomes and knowledge retention. A recent study demonstrated the effectiveness of team-based learning in enhancing student engagement and collaborative learning. 26

Balancing core subjects in the curriculum while introducing practical and clinical components is crucial. Research supports that a strong foundation in basic sciences is essential for medical students, forming the basis for clinical decision-making. Medical education literature often emphasizes integrating basic science and clinical practice to foster a deep understanding of medical concepts. 27 28

The call for a change in the assessment system aligns with the broader movement in medical education to shift from traditional summative assessments to formative assessments that promote continuous learning and skill development. Evidence suggests that formative assessments, such as objective structured clinical examinations (OSCE) and objective structured practical examinations (OSPE), enhance students' clinical skills and prepare them for real-world clinical scenarios. A systematic review found that OSCEs effectively measure clinical competence and provide valuable feedback for improvement. 29 Adequate faculty and teacher training are crucial for effectively implementing curriculum changes. The importance of faculty development in medical education is well-documented. Training programs for faculties can help them design and deliver innovative, student-centered teaching methods and assessments. Existing literature also emphasizes the role of faculty development in enhancing the quality of medical education. 30 The recommendation for a flexible curriculum with integrated projects aligns with individualized learning pathways in medical education. Personalized learning and integrating research projects or elective rotations can promote students' autonomy and passion for learning. The literature discusses the benefits of such flexibility, allowing students to explore their interests while meeting core learning objectives. 31

Addressing the concern about foreign cultural influence in medical education can be achieved through an India-centric approach that integrates local epidemiology, dietary guidelines, and cultural aspects into the curriculum. Studies have explored the impact of culturally adapted medical education on enhancing cultural competence and patient-centered care. 32 Doubts and concerns regarding changes in medical education are not uncommon, highlighting the need for clear communication, transparency, and continuous feedback mechanisms in the curriculum development process. Literature underscores the importance of involving stakeholders, including students and faculties, in curriculum design and revision. 33 Concerns about reservation policies in postgraduate entrance examinations raise essential questions about equitable access to medical education. Research has explored the impact of reservation policies on medical education and healthcare delivery. Studies have shown that affirmative action policies can enhance diversity in the medical workforce, improving healthcare access for underserved populations. 34 35

Efforts to bolster medical education necessitate the removal of redundant topics and outdated content, thereby optimizing the curriculum to enable students to concentrate on fundamental knowledge and skills. This study offers insights into the perceptions of faculties and students concerning curricular redundancy, pinpointing areas necessitating improvement. These improvements encompass transitioning from traditional lecture-based classes to active learning methodologies that integrate advancements in medical science and technology, including emerging domains such as epigenetics, genomics, and artificial intelligence, alongside embracing interdisciplinary education.

A forward-looking curriculum is indispensable, emphasizing the doctor–patient relationship, mental health awareness, hospital management, and practical proficiency. The adoption of a formative assessment system emerges as a pivotal domain conducive to reinforcing skill-based active learning.

## Limitations

The limitations of our research, primarily stemming from the constraints of sample size attributed to the use of electronic media and time constraints aligned with respondents' attention span, are noteworthy. The inability to differentiate between faculty and students' feedback for subgroup analysis further compounds these limitations. 36 It is imperative to acknowledge these constraints when interpreting and applying the findings of this study to inform decisions regarding the redundancy in the medical undergraduate curriculum and the enhancement of medical education in India. Subsequent research endeavors should aim for larger, more representative samples and employ additional data collection methods to address these limitations comprehensively and attain a more nuanced understanding of the subject matter.

## Conclusion

This study highlights the critical imperative for a flexible curriculum with integrated projects and prioritizing region-centric content. Addressing local epidemiology, dietary guidelines, and cultural nuances is indispensable for effectuating curriculum modifications successfully.
